# Some Common Medicinal Plants with Antidiabetic Activity, Known and Available in Europe (A Mini-Review)

**DOI:** 10.3390/ph15010065

**Published:** 2022-01-04

**Authors:** Monika Przeor

**Affiliations:** Department of Gastronomy Science and Functional Foods, Poznań University of Life Sciences, 60-637 Poznań, Poland; monika.przeor@up.poznan.pl; Tel.: +48-61-846-63-30

**Keywords:** antidiabetic, hypoglycaemic activity, medicinal plants, white mulberry, fenugreek, cinnamon, ginseng, ginger, common bean, diabetes

## Abstract

Diabetes is a metabolic disease that affected 9.3% of adults worldwide in 2019. Its co-occurrence is suspected to increase mortality from COVID-19. The treatment of diabetes is mainly based on the long-term use of pharmacological agents, often expensive and causing unpleasant side effects. There is an alarming increase in the number of pharmaceuticals taken in Europe. The aim of this paper is to concisely collect information concerning the few antidiabetic or hypoglycaemic raw plant materials that are present in the consciousness of Europeans and relatively easily accessible to them on the market and sometimes even grown on European plantations. The following raw materials are discussed in this mini-review: *Morus alba* L., *Cinnamomum zeylanicum* J.Presl, *Trigonella foenum-graecum* L., *Phaseolus vulgaris* L., *Zingiber officinale* Rosc., and *Panax ginseng* C.A.Meyer in terms of scientifically tested antidiabetic activity and the presence of characteristic biologically active compounds and their specific properties, including antioxidant properties. The characteristics of these raw materials are based on in vitro as well as in vivo studies: on animals and in clinical studies. In addition, for each plant, the possibility to use certain morphological elements in the light of EFSA legislation is given.

## 1. Introduction

Diabetes mellitus is a serious metabolic disorder. The reason for chronic hyperglycaemia can be: (1) a lack of production of an adequate amount of insulin or (2) the impossibility of peripheral tissues to react to the presence of insulin [1].

Diabetes mellitus spreads rapidly. In 2019, 9.3% of the global adult population were found to be diabetic [2]. The International Diabetes Federation in 2017 reported that the risk of type 2 diabetes concerned 352 million people. According to health forecasts, 439 million adults will have been affected by diabetes by the year 2030 [3]. Mortality from diabetes in 2010 ranged from 6% of all deaths in Africa to 15.7% of all deaths recorded in North America [4], and a meta-analysis of studies conducted during the COVID-19 pandemic showed that diabetes increases mortality in patients with COVID-19 [5]. The need to slow down the development of diabetes seems to be significant for maintaining the homeostasis of society in the world, also in view of the possibility of new pathogens and diseases. The overlapping of many diseases can result in irreversible health damage.

Historical reports and centuries-old cultural traditions show that some plants can be an alternative to standard pharmacotherapy or, at least, help with treatment or have a preventative effect. Modern science is very eager to verify these properties by analyzing the so-called medicinal plants for the presence of valuable bioactive compounds, including antioxidants, and the resulting interesting potential health properties [6,7,8,9,10,11,12,13,14]. In order for them to be introduced into the diet, their purity in terms of toxic compounds must also be taken into account [15,16].

There are different possible ways of introducing such plants into the diet. In Table 1, two different ways are shown.

The first way is to eat them on our own in a basic or modified form or in a slightly changed state of matter. The second way to use raw plant materials with antidiabetic activity is to change the matrix of the given substance, i.e., producing dietary supplements or designing foods containing a given raw material and giving them strictly defined characteristics, i.e., producing so-called functional food. This type of food is aimed at people with elevated blood glucose levels and used in the manufacture of food for obese patients. Technologists design such food products [17], and consumers increasingly seek such food [18]. In recent years, there has been a considerable and observable emphasis, or at least fashion, as to the maintenance of full health and vitality even in old age.

Although oral administration is the most common and simplest (for an average patient) way of introducing such raw materials into the human gastrointestinal tract, other ways of administration of such plants are also being analyzed [19,20].

There are many conventional pharmaceuticals for diabetes available on the market; however, their prices and the possible side-effects of long-term intake force the search for plant substitutes.

## 2. Medicinal Plants with Antidiabetic Activity

Many plants are rich sources of bioactive compounds with specific pharmacological properties, and they do not cause undesirable side effects [21,22]. For many years, the communities of developing countries have placed high hopes on these plant treatments, and the use of cheap medicinal plants instead of drugs to treat diabetes is common there [23]. At present, developed countries are also more inclined to adopt such solutions.

Medicinal plants contain various phytoconstituents (e.g., terpenoids, saponins, flavonoids, carotenoids, alkaloids, glycosides) with antidiabetic activity [14,24,25,26]. The complex plant matrix is a carrier of many phytoconstituents, which determines the specific interaction of these compounds; this is, however, difficult to reproduce and brings health benefits [27]. Chan et al. [28] noted in their work that antidiabetic ingredients are definitely most frequently found in leaves (more than 35% of the analyzed plants), while in other morphological elements are 3 times less frequent (about 10% each). 

Based on the literature review [28], six general antidiabetic mechanisms of active pharmaceuticals can be specified:

1. Alteration of glucose metabolism: inhibition of renal reabsorption of glucose [29], inhibition of β-galactosidase [30], inhibition of β-glucosidase [30,31], inhibition of α-amylase [30,31], glycogenesis stimulation [32], hepaticglycolysis stimulation [32], starch conversion to glucose inhibited [30,31];
2. Hypolipidemic effect: lipid peroxidation decrease [33];
3. Pancreatic effect: effect of regeneration/repairing of β-cells [34], protective effect on β-cells [35], effect of increasing number and/or size of cells in Langerhans islets [34], insulin resistance reduction [36], insulin secretion stimulation [36,37], inhibition of degradative processes of insulin [36];
4. Antioxidative effect: protection against the effects of oxidative stress responsible for β-cell dysfunction [38] by scavenging free radicals, reducing H_2_O_2_ formation, inhibition of ROS production, modulation of enzymes (cyclooxygenase, microsomal monooxygenase, NADH oxidase, xanthine oxidase, lipoxygenase, succinoxidase) [39], regulation of antioxidant:oxidant balance in cells [33], induction of enzymes (glutathione peroxidase, catalase, superoxide dismutase) [33], improvement of antioxidant capacity in plasma [33];
5. Diabetes complication treatment: inhibition of pro-inflammatory pathway of NF-κβ, resulting in vascular complications [40];
6. Insulin-like effect.

Alternation of glucose metabolism is the most common one. The use of medicinal plants based on this most common scheme is mainly based on supporting pancreatic function—increasing insulin secretion or decreasing intestinal glucose uptake [21,23].

Therefore, inhibitors that interfere with digestive enzymes, which are responsible for the hydrolysis and absorption of macroelements, are important. The problems with the maintenance of normal glycaemia can be reduced by inhibition of enzymes digesting carbohydrates: pancreatic α-amylase (breakdown of polysaccharides to oligosaccharides and disaccharides) and brush border α-glucosidase (breakdown to monosaccharides) [41]. Some studies suggest that the most significant natural inhibitors, due to their presence in many antidiabetic plants, are terpenes, saponins, and polyphenols [41,42,43,44]. The literature presents many medicinal plants with antidiabetic or antihyperglycaemic activity, used in different regions of the world. Recently, Salehi et al. [21] indicated 703 plants as being α-amylase and/or α-glucosidase inhibitors and most often discussed in the literature.

This review describes some antidiabetic medicinal plants (white mulberry, fenugreek, cinnamon, common bean, ginger, and ginseng) widely available, quite cheap for the average consumers, and trusted by Europeans, especially in Central Europe.

### 2.1. White Mulberry (Morus alba L.)

Mulberry is a fast-growing, deciduous plant from the *Moraceae* family that is found at various geographical latitudes, i.e., in climates from tropical to moderate [45].

White mulberry originates from China, Japan, and India, and historical sources have revealed that all its parts, i.e., fruits, leaves, and bark, were already used in medicine in 3000 B.C. It was brought to Europe in the 11th century with silkworm caterpillars. It is also very common in other countries of Asia, Europe, and America [46]. White mulberry is also grown in Europe due to low agrotechnical requirements, relatively easy and cheap cultivation, and the possibility of using it in the food industry [47,48,49,50].

Mulberry leaves contain nutrients such as proteins, lipids, carbohydrates, fiber, β-carotene, xanthophylls, vitamin C, and complex vitamins, including folic acid, vitamins D and E, β-sitosterol, and minerals [11,14,51]. Mulberry leaves are also rich in valuable compounds of pharmacological activity, including polyphenolic compounds such as quercetin 3-(6-malonyl-glucoside), kaempferol 3-(6-malonyl-glucoside), rutin, morcetin, isoquercetin, astragalin, and other glycosides, tannins, and coumarins, as well as phenolic acids [14,25,52]. The most common biologically active compounds present in white mulberry and defined as probably antidiabetic are shown in Table 2.

Numerous scientific reports have proven that chemical substances, including polyphenolic compounds, contained in white mulberry leaves demonstrate antiradical activity as well as chelating and reducing properties [11,25,73,74,75]. Thus, extracts of mulberry fruits may prevent liver cancer [76], and leaf extracts lower postprandial glucose [77] or are used in antidiabetic treatment [78,79] and show antibacterial activity [80]; white mulberry can inhibit atherosclerosis [81] due to its antioxidant activity [74,82]. In Chinese medicine, the brews are used in the treatment of colds, sore throats, and toothache and also in liver protection and for the purposes of lowering blood pressure [83,84]. Moreover, white mulberry extracts are used in the treatment of skin discoloration and its regeneration [85].

Antidiabetic activity is strongly related to the anti-obesity effect of *Morus alba*. There are preclinical studies that present the mechanisms responsible for the anti-obesity effects of white mulberry. They include:

- Inhibition of digestive enzymes, i.e., pancreatic lipase, cholesterol esterase [86], pancreatic α-amylase (weak effect) [87], intestinal α-glucosidase [74];
- Adipocyte differentiation [88,89];
- Influence on the appetite [90];
- Regulation of lipid metabolism: improvement of lipid profile [91,92];
- Improving the oxidative status of the organism [93,94,95].

At the same time, it is difficult to find clinical studies that undertake the issue of this relationship [89].

Mulberry leaves are considered an important raw material, having antidiabetic and hypoglycaemic activity [53,96,97]. This is related to the presence of alkaloids, including 1.5-dideoxy-1.5-imino-D-sorbitol (DNJ), terpenes, and steroids [53,54,55]. *Morus alba* has a natural flavonoid—morin—that is an activator and sensitizer of the insulin receptor stimulating the metabolic pathways [21]. Morin can also reduce endoplasmic reticulum stress (this factor, combined with hyperglycaemia, largely contributes to the pathogenesis of type 2 diabetes) in diabetic rats [56]. 

A study conducted on diabetic rats demonstrated that a 5-week-long administration of mulberry leaf extract reduced blood glucose levels by 5% [19]. One of the substances responsible for such activity is DNJ, inhibiting α-glucosidases, e.g., saccharase, isomaltase, maltase, and, primarily, glucoamylase; it is responsible for polysaccharides’ decomposition to glucose, absorbed by the intestinal villi [54,75]. Moreover, white mulberry also belongs to the group of α-amylase inhibitors [98].

Oral administration of mulberry leaf powder in humans in the amount of 0.8 g and 1.2 g considerably inhibited postprandial glycaemia and insulin secretion [99]. Therefore, mulberry leaves are used for Mon Tea—an antidiabetic tea prepared in Korea, Japan, and Chile [52]. Such teas and numerous pharmaceutical preparations with mulberry leaf extract additions are also common in Europe. Hansawasdi and Kawabata [100] reported that about 1 g of mulberry leaves is needed for the preparation of 100 mL of tea brewed in water of a temperature of 98 °C for 3–5 min in order to inhibit α-glucosidase activity. In a group of diabetic rats treated with polysaccharides isolated from *Morus alba* fruits for 7 weeks, reductions in the FBG, FSI, and oral glucose tolerance were observed compared to a group in which a diet without these polysaccharides was used. Additionally, polysaccharides from mulberry fruits used in the diet contributed to the improvement of pancreatic tissues in diabetic rats [101]. In turn, a study conducted on diabetic mice fed for only 28 days on a mixture of mulberry leaves and oat bran (6 g per kg of body weight; ratio 1:1) showed significantly higher hypoglycaemic activity (defined as lower levels of FBG and lower PPG_60min_ in oral glucose tolerance tests) and more significant inhibition of aspartate transaminase and alanine transaminase activities compared to mice fed exclusively on mulberry leaves or exclusively on bran (in the same amount) [102].

Importantly, studies show that white mulberry leaves show no acute toxicity (LD_50_ >15.0 g per kg of body weight), no subacute toxicity (NOAEL = 7.5 g per kg of body weight per day), and no genotoxicity, which makes them appear to be a safe food ingredient [103]. Currently, however, according to the EU novel food catalog, the only morphological element of white mulberry *Morus alba* recognized as a safe food ingredient in the European Union is its fruit (Table 3) [104].

### 2.2. Fenugreek (Trigonella foenum-graecum L.)

Fenugreek is an annual herbal plant with fine seeds from the *Leguminosae* family. In its natural state, fenugreek is found in the Mediterranean area as well as in India and China. It is cultivated mainly as a forage plant. Both the seeds and leaves of fenugreek appear in literature as an ingredient of food and as medicine [105]. 

The high levels of protein, vitamins, and mineral compounds mean that germinated fenugreek seeds are a valuable component of a so-called healthy diet. The seeds of fenugreek contain mucous polysaccharides—galactomannans (25–45%), proteins (43.8%; mainly tryptophan and lysine), free amino acids (mainly 4-hydroxyisoleucine and histidine) [106], fats (7.9%) [107], steroid saponins, alkaloid—trigonelline, coumarins, flavonoids, sterols, lecithin, and choline as well as nicotinic acid (formed from the breakdown of trigonelline during roasting) and minerals [108]. Fenugreek leaves are a source of β-carotene (0.019%), ascorbate (0.22%), iron, calcium, and zinc [106]. In the group of fenugreek polyphenol compounds, rhaponticin and isovitexin are believed to be the most bioactive [109].

The presence of these compounds and their interaction have a positive effect on the course of many diseases and help to protect against their occurrence. Both in vitro and in vivo studies have been conducted on the therapeutic or prophylactic role of fenugreek.

Nutraceutical properties of fenugreek include, i.a., blood purification; sweat-inducing effects, supporting the removal of toxins; cleaning the lymphatic system; maintaining mucous membranes in good condition; removing excess mucus from the throat; relieving colds, bronchial problems, flu, asthma, rhinitis, constipation, sinusitis, pneumonia, and laryngitis [105]. The use of fenugreek seeds in supporting the treatment of neurodegenerative diseases such as Parkinson’s disease has also been analyzed [110]. 

With respect to the gastrointestinal tract, it was observed that dried or germinated fenugreek seeds, or a pap made of them, stimulate appetite and digestion and are used in alimentary tract disorders: dyspepsia, tympanites, gastritis, and liver diseases. They are also used as a raw material with expectorant activity in upper respiratory tract diseases. In traditional medicine, hot compresses made from fenugreek seeds (cataplasms) are used in the treatment of local inflammatory conditions of the skin and subcutaneous tissue, e.g., furuncles, abscesses, and ulcerations [111].

*Trigonella foenum-graecum* is a valuable raw material in the regulation of the lipid profile. In studies [112] with Wistar rats with obesity induced by a high-fat diet, reduction in body weight gain, body mass index, blood glucose, white adipose tissue weights, and serum insulin were observed. In clinical studies, a 30-day administration of 25 mg of fenugreek seed powder solution significantly helped in the area of dyslipidemia in newly diagnosed type 2 diabetic patients [1].

Fenugreek has antioxidant properties, resulting from the presence of, i.a., gallic acid, protocatechuic acid, catechin, gentisic acid, chlorogenic acid, and vanillic acid [106]. In a DPPH radical test, the value of IC_50_ for a *Trigonella foenum-graecum* seed methanol extract was determined at 350 µg/mL, while in a ABTS IC_50_ cation radical test, it was 117 µg/mL [113]. 

Fenugreek is considered to be one of the Indian plant species exhibiting antidiabetic activity [21,24,59]. The hypoglycaemic or antidiabetic activities of fenugreek leaves have been the subject of many studies. The most common compounds of fenugreek identified as probably antidiabetic are shown in Table 2.

The antiglycaemic activity of fenugreek probably results from the synergistic activity of various chemical compounds. Some research explains that the presence of galactomannans means that consumption of fenugreek seeds in the form of pap delays stomach emptying, moderates carbohydrate absorption, and inhibits glucose transport [57]. It has been proposed that the mucilage contained in the raw material covers the intestine diffusion layer and thus retards nutrient absorption, including carbohydrates [114]. It has also been demonstrated that an effect of *Trigonella foenum-graecum* extract activity involves an increase in the amount of erythrocyte insulin receptors and thus peripheral glucose consumption [57]. Hypoglycaemic activity may be related to the normalization of gluconeogenic enzymes and a decrease in glycolytic enzyme activity [111]. In turn, Broca et al. [58] conducted a study on rats with induced diabetes and demonstrated that 4-hydroxyisoleucine was the active component causing the hyperglycaemia reduction. In this study, the administration of 4-hydroxyisoleucine to sick animals for 6 days resulted in a glycaemia reduction from 163.5 to 143.6 mg/dL [58]. This amino acid has been identified as the main active component of fenugreek seeds by many other researchers [115]. 4-Hydroxyisoleucine inhibits insulin secretion in a wide concentration range, which contributes to a decrease in blood glucose levels. Apart from lowering glucose levels, fenugreek seeds also cause a reduction in TC [114].

There are also some reports suggesting that the hypoglycaemic properties of *Trigonella foenum-graecum* result from its high content of dietary fiber (up to as much as 30%), especially its insoluble fraction [116]. Steroid saponins from *Trigonella* are also indicated as bioactive compounds responsible for the antidiabetic effect of this spice [59], and their amount shows wide variability among the fenugreek genotypes [117]. 

In rats with induced diabetes, it was observed that consumption of ethanolic fenugreek seed extract (0.25 and 0.5 g per kg of body weight) for 14 days significantly reduced serum glucose compared to the control group. The level of changes was very similar to that caused by glibenclamide—a drug used for the purposes of attenuation of serum parameters in diabetics [118]. 

The antidiabetic potential of fenugreek seeds extract was analyzed in a 4-week study with streptozocin-induced diabetic Sprague-Dawley rats. The dose of 100 mg per kg of body weight significantly reduced blood glucose, reduced levels of liver enzymes (aspartate aminotransferase and alanine aminotransferase), and reduced triglycerides. Moreover, mild protection of hepatic, renal, and pancreatic tissues after fenugreek administration was observed [20].

In clinical trials, on the other hand, consumption of *Trigonella* seed extract, enriched in 40% of furostanolic saponins for 30, 60, and 90 days, resulted in a 6.69%, 10.31%, and 21.98% reduction of FBG, respectively, a 13.7%, 20.6%, and 30.4% reduction in postprandial blood glucose levels, and a reduction in glycosylated hemoglobin levels (but not significant) [119]. In studies by Singh et al. [120], 20 patients with type 2 diabetes took 5 mg of glipizide per day (Group A) for 12 weeks, 20 patients took 500 mg of fenugreek seed extract twice a day (Group B), and 20 patients took 2.5 mg of glipizide + 500 mg of fenugreek seed extract per day (Group C). In all groups, a significant decrease in FBG (A-33.97% > C-29.96% > B-24.62%) and glycated hemoglobin (A-12.98% > C-10.62% > B-9.38%) was observed; in Groups B and C, a significant decrease in TC (respectively: −5.66% and −3.87%), plasma triglycerides (−17.23% and −11.34%, respectively) and LDL cholesterol (−4.15% and −2.68%, respectively) could be noted. On this basis, it was concluded that fenugreek therapy (alone or in combination with drugs) significantly improved glycaemic and dyslipidemic control.

In terms of inhibition of pancreatic lipase, the ethanol extracts of fenugreek compared to the ethanol extracts of quinoa showed 10-fold higher inhibitory activity. In turn, the levels of α-amylase inhibition by these two raw materials were significantly lower than those obtained in tests with pancreatic lipase and were similar to each other—mild inhibition (24.8% for quinoa and 27.3% for fenugreek concentrated extracts) [41].

Fenugreek allergenicity analyses, including the level of specific IgE antibodies, have shown that fenugreek has many potential allergens and a high level of cross-reactivity with peanuts [121]. Moreover, this plant has a probable teratogenic and abortive effect and changes hematology and blood biochemistry [122]. According to EFSA, only for fenugreek seeds, a safety assessment is not required (Table 3) [104].

### 2.3. Ceylon cinnamon (Cinnamomum zeylanicum J.Presl)

Ceylon cinnamon belongs to the *Lauraceae* family; it originates from Ceylon but is cultivated in various regions of southern Asia and North America. The raw material is bark (*Cinnamomi cortex*) without the internal layer, the so-called primary bark. Ceylon cinnamon bark contains 0.5% to 4.0% oil, depending on the origin of the raw material. The main components of the oils are as follows: cinnamaldehyde (65–75%), cinnamyl acetate and eugenol (ca. 5% in total), and β-caryophyllene (up to 4%). Moreover, the bark contains polysaccharides (mucilage), phenolic acids (cinnamic acid and its derivatives), oligomeric proanthocyanidins, diterpenes, and others [123].

For centuries, cinnamon has been used in Chinese homes as a spice and also as a traditional Chinese remedy for cold and flu [124]. Historically, it has also been known for its antibacterial, antifungal, and carminative properties [125,126].

The antioxidative and antibacterial activity of an extract derived from cinnamon has been demonstrated in recent years [127]. Among the best-known herbs and spices in terms of antioxidant content, researchers indicate that cinnamon (77 mM per 100 g of antioxidant) has less antioxidative properties than only several other plants, which include allspice, cloves, and peppermint [63]. Ethanol extracts from cinnamon bark in the ABTS cationic radical test reached the value of 525.85 µM Trolox equivalent per g of dry weight, 87.45%—in the DPPH radical test, and 637.00 µM Trolox equivalent per g of dry weight in the FRAP test [128]. This is important in reducing the oxidative stress of patients.

Studies have also demonstrated that cinnamon bark in doses of 1–6 g per day causes a reduction in TG, TC, and LDL fraction in patients with type 2 diabetes [63]. This is probably caused by the presence of a methylhydroxychalcone polymer (MHCP) stimulating, almost like insulin, glucose uptake by adipocytes [61]. In the study by Jarvill-Taylor et al. [61], MHCP activated insulin receptor autophosphorylation and, thus, glucose uptake and glycogen synthesis. Thus, a synergism between MHCP and insulin was observed since the concurrent application of both substances induced a considerably better response than the sum of responses resulting from their separate application. Another study [62] conducted on rats suggested the significant importance of cinnamaldehyde in antiglycaemic and antilipemic activity. 

Antidiabetic activity was analyzed in the studies on diabetic rats [129]. It was observed that administering 200 mg of ethanolic extract of *Cinnamomum zeylanicum* per kg of body weight to animals once a week for 4 weeks had a hypoglycaemic effect. In this study [129], blood glucose (from 257.0 to 122.9 mg/dL after 4 weeks) and glycosylated hemoglobin levels were reduced.

Mirfeizi et al. [130] noted in a randomized controlled trial that the introduction of cinnamon into the diet of patients with type 2 diabetes mellitus, in a glucose load test, reduced the glycaemia after 90 min to 224 mg/dL, while without the use of cinnamon in the same patients, the glycaemia after 90 min was 270 mg/dL. On the other hand, Vafa et al. [131] observed, by administering 3000 mg of cinnamon powder daily for 8 weeks to 44 patients, a reduction in insulin serum levels by 12.87 mIU/dm^3^ and a reduction in FBG by 0.45 mg/dL.

Santos and Silva [63] indicate six pathways improving serum parameters and fat loss:

1. Cinnamon fiber delays the emptying of the stomach;
2. Eugenol from cinnamon acts as an inhibitor of α-glucosidase in the intestines;
3. In the myocyte, there is an improvement of insulin receptor phosphorylation, synthesis, and translocation of GLUT-4 to glucose uptake and, therefore, an increase of glycogen;
4. Cinnamaldehyde provides sympathetic actions; increased noradrenaline may increase the heart rate and thermogenic influence on brown adipose tissue;
5. The proposed mechanism of body fat loss across cinnamon intake occurs from the UCP1 activation in the mitochondria of brown adipose tissue and greater PPAR-α expression in white adipose tissue and, consequently, increases β-oxidation by means of enzymatic action of acyl-CoA oxidase;
6. Expected improvement of glycaemic, lipid, and antioxidant parameters.

Moreover, according to the research, bioactive compounds from Ceylon cinnamon show potentially beneficial activity in the treatment of cancer [132], inflammation, immunomodulatory diseases [133], and wound healing [134]. Ceylon cinnamon supplementation decreased the blood pressure of diabetes patients in clinical trials while not affecting body weight, body mass index, and waist circumference [135]. Cinnamaldehyde, found in cinnamon, has been indicated as promising and safe for the treatment or prevention of Alzheimer’s disease [123].

Due to positive premises from experimental studies, cinnamon application as a factor adjunctive to carbohydrate metabolism also seems to be an interesting alternative in functional food supporting the treatment of diabetes.

According to the EFSA, *Cinnamomum zeylanicum* bark of the branches is used as food and leaves are used as food supplements in Europe. A request for the oil of the leaves is being processed (Table 3) [104].

### 2.4. Ginger (Zingiber officinale Rosc.)

Ginger is one of the oldest spice and medicinal plants [136]. Common ginger, which belongs to the *Zingiberaceae* family, is a herbaceous plant with a strong rhizome divided into tuberous sections. It has sterile shoots up to 1.5 m, with evenly narrow lanceolate leaves 5–30 cm long and 8–20 cm wide. It probably originated in Southeast Asia, and it is cultivated in many tropical regions, including Africa, China, India, and Jamaica [67]. In Europe, ginger is very widespread and is often used in combination with Far East cuisine.

Ginger is rich in essential oils, the amount of which, in the rhizome, ranges from 1% to 3%. Among more than 50 identified essential oils, the ones to be mentioned in particular are monoterpenes (felandrene, camphene, 1,8-cineol, geranial, citral, terpineol, borneol) and sesquiterpenes (ar-curcumen and α-zingiberene 30–70%, β-sesquifelandrene 15–20%, β-bisabolene 10–15%, zingiberol). Gingerols are responsible for the spiciness of fresh ginger, while their dehydrated forms, shoagols, are responsible for the spiciness of dried ginger. In addition, ginger also contains diarylheptones, diterpenes, and monoacyl-digalactosylglycerols [65]. Due to the popularity of the plant in nutrition, special attention is paid to the variability of biologically active compounds in ginger as a result of drying. Freeze-dried and infrared and intermittent microwave-convection drying material has better antioxidant properties, higher retention of gingerols, phenolics, and flavonoids than air-dried (60 °C) or microwave-dried slices of ginger [137].

In the past, in traditional medicine, ginger was used as an ingredient with carminative, expectorant, and astringency properties [138]. The studies conducted so far indicate that the beneficial qualities of ginger rhizomes are due to, among other things, its hypoglycaemic, hypocholesterolemic, antiarthritic, antirheumatic, and antioxidant activity [139,140,141,142]. The use of ginger extracts reduced a high level of TC in rabbits who were on a 10-week high-fat diet, proving the antihyperlipidemic properties of ginger [143]. Ginger is also known for its analgesic and anti-inflammatory qualities, which is evidenced by its inhibitory effect on prostaglandin synthesis. It was also demonstrated that ginger contains components with pharmacological properties that imitate anti-inflammatory drugs. In in vitro studies, aqueous extracts had a greater inhibitory effect on lipoxygenase than diclofenac (58% vs. 52%), while in in vivo models, they beneficially reduced edemas in rats and demonstrated identical effects to indomethacin (a strong anti-inflammatory and analgesic agent) in the reduction of NOx. The most potent active compounds were 6-paradol, 6-shogaol, and 1-dehydro-6-gingerol [66]. The effects of ginger essential oils, in the amount of 28 mg per kg per day, prevented chronic arthritis, comparable to 17-β-estradiol, in an animal model [144]. Jafarzadeh and Nemati [145] identified many possible mechanisms of action of ginger active ingredients with immunomodulatory, anti-inflammatory, and antioxidative potential in the context of multiple sclerosis treatment. On the other hand, ginger protects tissues from radiation [146] and shows chemopreventive effects against some skin and breast cancer [147]. In Middle Europe, many people use ginger during the fall and winter season as an important element of their diet to prevent infections and to treat upper respiratory tract infections.

Ginger has an antidiabetic effect, which was demonstrated in several studies on different models. Conducting studies on rats with induced diabetes, feeding them with ginger extract in the amount of 4 mL per kg of body mass pr day per 6 weeks significantly reduced blood glucose compared to sick animals. It concerned animals fed with ginger, both before and after inducing diabetes [65,141]. The consumption of ginger juice in the amount of 4 mL per kg per day for 6 weeks flattened the blood glucose and insulinemia curve in the glucose tolerance test in the group of diabetic rats [148]. Aqueous ginger extract (in the amount of 100–500 mg per kg) administered daily for 30 days to rats increased the activity of glycolytic enzymes and had an antihyperglycaemic effect [149]. Ethanolic ginger extract included in an animal diet in the amount of 200 mg per kg for 30 days reversed hyperglycaemia and improved the activities of extra- and intra-mitochondrial enzymes, resulting in a nephroprotective effect [150]. Ginger increases insulin sensitivity, protects pancreatic β-cells, and reduces oxidative stress in rodents [151]. Model studies in L6 mouse myoblast and myotubes showed that the main components responsible for the antidiabetic potential of ginger are shoagol and gingerol [152,153].

Clinical research has confirmed the antidiabetic properties of ginger. In a group of newly diagnosed obese (BMI > 30 kg/m^2^) patients with diabetes, El Gayar’s team [154] showed that daily consumption of 3 capsules, each containing 600 mg of ginger (dried rhizome) powder for 8 weeks, resulted in a significant (*p* < 0.001) reduction in BMI (−0.54 kg/m^2^), HbA_1c_ (−1.11%), FBG (−51.15 mg/dL), FSI (−7.88 mIU/L), TC (−31.10 mg/dL), and LDL cholesterol (−17.70 mg/dL). In Khandouzi’s study [155], consumption of a slightly higher dose of ginger powder (2 g per day for 12 weeks) had similar effects (FBG: −19.41 mg/dL; HbA_1c_: −0.77%; apolipoprotein B: −12.45 mg/dL). Iranian patients with type 2 diabetes who consumed 3 g of ginger powder in the form of capsules showed a significant improvement in diabetic parameters (serum glucose: −19.41 mg/dL; HbA_1c_: −0.77%; SI: −1.46 µIU/mL; insulin resistance: −16.38; high-sensitive CRP: −2.78 mg/dL) compared to the control group of patients [156]. On the other hand, in another group of patients with diabetes mellitus, ginger powder added to the diet in the amount of 3 g per day decreased only the SI but did not affect FBG and HbA_1c_ [157].

**Figure 1:**
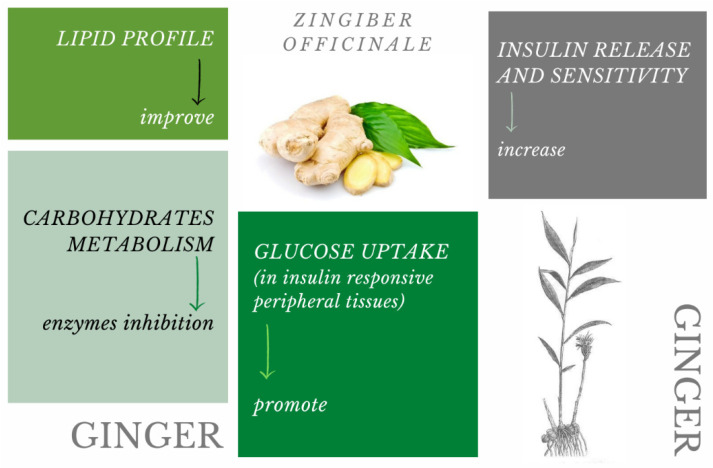
The ways in which the active ingredients in ginger affect glycaemic control [158].

The ways in which the active ingredients in ginger affect glycaemic control are shown in the diagram (Figure 1).

Due to its properties, ginger can be used in the production of medicinal functional food because of its effectiveness and non-toxicity [139]. The FDA approved ginger as ‘GRAS’ plant material at a dosage of < 4 g per day. However, at doses of >6 g of ginger per day, researchers reported some side effects (heartburn, mild diarrhea, abdominal discomfort) [147]. In Europe, ginger is highly trusted by consumers and is easily accessible on the market.

### 2.5. Common Bean (Phasolus vulgaris L.)

Common bean is a member of the *Fabaceae* bean family, and the pericarp of *Phaseoli pericarpium* is a medicinal raw material. Common bean originated from Europe and Western Asia, and it is widely cultivated in North America, mostly because of its culinary value [159].

The nutritional value of bean seeds is due to their high content of protein, starch, B vitamins, and minerals. Important non-nutritional components of bean seeds are polyphenolic compounds, which are most responsible for the antioxidant properties of those raw materials [160,161]. Beans are among the most popular leguminous plants grown for food purposes in Poland and other countries. The usable parts of the bean are the unripe pods, called string beans, and the dry ripe seeds.

In addition to hypoglycaemic compounds such as guanidine derivatives (amino-β-guanidino-isovaleric acid), phaseoloside, and chromium salts, common bean also contains amino acids, choline, trigonelline, allantoin, pipecolinic and traumatic acid, and flavonoids [161,162]. The main activity of the bean pericarp is its diuretic effect and protecting effect in the case of kidney dysfunction resulting from hyperglycaemia [163].

The antidiabetic activity of beans has been demonstrated in animal studies. As a result of feeding rats with induced diabetes with a bean extract (200 mg per kg, 28 days), an increase in the ability of GLUT-4 to regulate glucose utilization (increase in GLUT-4 content in skeletal muscle) was observed [164]. In the group of streptozotocin-induced diabetic rats with a diet enriched with cooked common bean (100 mg/kg), FBG was reduced by 25% after 2 weeks and by 35% after 4 weeks [163]. Aqueous bean extract administered to animals for 40 days in slightly higher amounts (100 mg and 200 mg per kg) had similar effects (FBG −25% and −50%, respectively) and resulted in a significant reduction of TC and TG, while in the amount of 200 mg per kg, the extract produced similar medicinal effects to glibenclamide [165]. Studies conducted simultaneously on healthy and sick rats showed that the introduction of bean (300 g per kg) into the diet of animals treated with glibenclamide undoubtedly reduced the dose of the drug necessary to improve glycaemia [166]. In addition, common bean extract inhibited the adipogenesis of 3T3-L1 adipocytes and reduced the lipid content in adipocytes (−20.71%) in vitro [167]. 

The knowledge acquired so far on the properties of common bean may be helpful in the treatment of diabetes, especially in kidney inflammation and other nephropathies that may be a consequence of hyperglycaemia.

### 2.6. Ginseng (Panax ginseng C.A.Meyer)

The root of this relict perennial from the *Araliaceae* family has been known in medicine for centuries. The description of its qualities can be found in the famous, ancient book of medicines „Shennong bencao” (XI century BC), but in Europe, it first appeared on the table of King Louis XIV [168]. The name ginseng stems from the shape that the root takes, i.e., the human body [169]. The raw material is dried roots collected in autumn from 4- to 6-year-old plants, with a minimum diameter of about 2 cm. Ginseng is found throughout China, from the slopes of the central Himalayas, through Korea and Japan, and to North America [170].

The main chemical components of this raw material include triterpene saponins (2–3%), panaxosides, and ginsenosides, which increase the mental and physical efficiency of the body and have an antistress effect. This raw material also contains polysaccharides, peptidoglycans (panaxans A-U), polyacetylenes, essential oil, sesquiterpene alcohols, sterols, flavonols, and phenolacids. Moreover, the activity of wild-growing ginseng is higher than plantation ginseng [171,172,173].

In Asian culture, ginseng has been used for centuries to reduce fatigue and mental weakness, to treat decreased libido, in stomach ulcers, in insomnia, to promote longevity, and to increase intelligence, as well as to improve eyesight, spleen function, and appetite [170,174,175,176]. Other possible uses of ginseng were also considered [177].

Currently, clinical studies have demonstrated that *Panax ginseng* root has extended preventive or even curative effects, protecting against cancer and nephropathy. Moreover, it improves blood circulation as well as protects against illnesses of the respiratory and nervous systems. It also improves liver activity, physical endurance, and bone metabolism and ensures the neutralization of changes in postmenopausal osteoporosis [178,179,180,181,182,183,184,185]. It is generally believed that ginseng improves metabolism and, thus, helps to improve and maintain good health, and, for this reason, it is an ingredient of dietary supplements [186].

Korean clinical research on a group of 72 patients with diabetes showed that consumption of a vinegar extract of *Panax ginseng* for 8 weeks at doses of 1500–3000 mg daily resulted in a notable reduction of HbA_1c_ (from −0.56% to −0.29%) and FBG (from −21.4 to −6.76 mg/dL) compared to the placebo group [187]. Additionally, the consumption of 2 capsules containing 30% of hydrolyzed ginseng extract daily for 8 weeks in the case of 23 diabetic patients resulted in a significant reduction of FBG and PPG_60min_, as well as a visible, but not statistically significant, reduction of PPG_30min_. The authors of the study concluded that ginseng slows down glucose absorption in the bowel or increases glucose intolerance at the stage of absorption [188]. Since CK and Rg1 ginsenosides increase glucose uptake in 3T3-L1 adipocytes with insulin [69], such an effect is possible. On the contrary, fermented ginseng in the amount of 2.7 g per day in the group of 40 people reduced PPG_120min_ (by 17.2%) and flattened the glucose curve (by 27.4%), with no effect on fasting glycaemia and insulinemia. The fermentation process applied could certainly improve the bioavailability of ginsenosides from the raw material, but the time of nutritional exposure was shorter than in other clinical studies [189]. On the other hand, in the group of 68 non-diabetic patients, consumption of 6 g of ginseng for 12 weeks did not affect insulin and insulinemia sensitivity [190]. Additionally, a shorter, 8-week introduction of ginseng (6 g per day) to the diet of obese women (50 individuals) reduced their obesity levels, regardless of the treatment used [191].

The effect of ginseng on the change and/or improvement of biochemical parameters in diabetic patients may be related to the interaction of several major bioactive compounds. Studies on mice on a high-fat diet showed that Rb1 ginsenoside weakens the symptoms of low insulin sensitivity and high glycaemia [192]. In the animals with induced diabetes, Rb1 also improves the lipid profile and lowers glycaemia and insulin sensitivity, becoming of interest for patients with coexisting liver disease [71]. On the other hand, Rg3 ginsenoside, formed by thermal degradation of other ginsenosides (e.g., during the production of red or black ginseng), shows the highest glucagon-like peptide-1 secretion from 15 ginsenosides analyzed by Kim’s team [70]. An increase in the expression of GLUT-1 and GLUT-4, which results in a greater uptake of glucose, has also been suggested as a possible factor in the therapeutic effects of ginseng against diabetes [193,194].

In Europe, ginseng is an available and well-known ingredient. It usually appears in a dried form, extracts, or dietary supplements [195]. According to EFSA, *Panax ginseng* was used as the food of food ingredient before the year 1997; thus, it is not a novel food product [104] (Table 3).

## 3. Conclusions and Future Perspectives

Because of their antidiabetic properties, various morphological elements of the above-described plants—mulberry, fenugreek, cinnamon, common bean, ginger, and ginseng—may be used as medicinal agents (Table 4). They can be administered to people in a variety of forms: basic, crushed, slightly processed, or as an ingredient of functional food. The status of these plants is described as non-toxic to the average patient, allowing them to be used as a substitute for conventional pharmacology. The antidiabetic properties of the analyzed plants have been confirmed by numerous in vitro tests and many pre-clinical in vivo studies published. Even though the results of the published papers are very favorable, the number of clinical trials is still disappointing. Increasing clinical trials, with the use of larger populations, is recommended. Moreover, there is a small number of trials considering mixed therapies (drugs and plant pharmaceuticals of natural origin). Plant-based diets are becoming more popular with the next generation of Europeans, also due to the ecological aspects. For this reason, future outcomes of clinical trials should indicate the optimal methods of introducing medicinal plants into the pharmacological treatment of diabetes mellitus and the optimization of doses and forms of plants in mixed therapies in order to avoid undesirable side effects. Simultaneously, the safety of the proposed therapies should be analyzed. The literature data also highlight many other activities of these plants, and the impact of bioactive compounds contained in them can be described as multi-fold. 

Despite their proven properties or traditional and historical medicinal successes, the presented raw materials are still only an adjunctive element in diabetes treatment and not the main agent in combating this disease. Undoubtedly, the biologically active substances contained in the discussed raw materials may considerably improve health status and prevent diabetes, especially type 2 diabetes. The fact that these raw materials are cheap, well known, and easily accessible on the European market should interest the inhabitants of developed countries.

## Figures and Tables

**Table 1:** Different possible ways of introducing medicinal plants into the diet.

How Can the Medicinal Plant Be Introduced into the Diet?		
First way		
Eat on your own	Basic form:	whole leaves
		whole seeds
		whole shoots
		whole fruits
	Modified form:	ground
		crushed
		dried
		cut
	Changed state of matter:	brew
		tea
		extract
Second way		
Change the matrix	Functional food products	
	Dietary supplements	

**Table 2:** The most common biologically active compounds identified as probably antidiabetic in medicinal plants.

Medicinal Plant	Biologically Active Compound Probably Responsible for the Antidiabetic Activity		Main Antidiabetic Mechanism of Action on Organism	Source
White mulberry *Morus alba* L.	1.5-dideoxy-1.5-imino- D-sorbitol (DNJ)	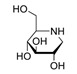	inhibition of α-amylase; inhibition of α-glucosidase; hypolipidemic; antioxidant	[53,54,55]
	morin	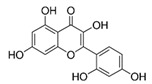		[21,56]
Fenugreek *Trigonella foenum-graecum* L.	galactomannans	-	decrease blood glucose concentration	[57]
	4-hydroxyisoleucine	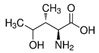		[58]
	saponins	-		[59]
	trigonelline + nicotinic acid	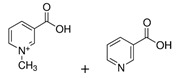		[60]
Ceylon cinnamon *Cinnamomum zeylanicum* J.Presl	methylhydroxychalcone polymer	-	elevation in plasma insulin; hypoglycaemic; hypocholesterolemic; stimulate glucose uptake by adipocytes;	[61]
	cinnamaldehyde	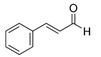		[62,63]
	eugenol	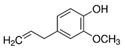		[64]
Ginger *Zingiber officinale* Rosc.	shogaol	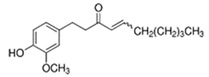	increase insulin level; decrease fasting glucose level	[65,66]
	gingerol	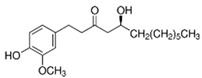		[66,67]
Common bean *Phaseolus vulgaris* L.	phaseolamin	-	hypoglycaemic; inhibit α-amylase activity; antioxidant; hypolipidemic	[68]
Ginseng *Panax ginseng* C.A.Meyer	ginsenoside Rg1	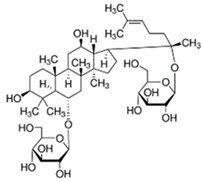	lowering blood glucose level; slows down glucose absorption; obesity reduction; increase in the expression of GLUT-1 and GLUT-4	[69]
	ginsenoside C-K	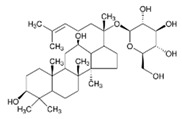		[69]
	ginsenoside Rg3	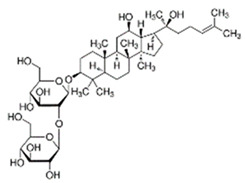		[70]
	ginsenoside Rb1	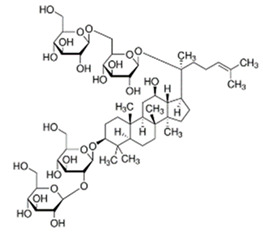		[71]

Source of chemical structures in this table: sigmaaldrich.com accessed on 13 December 2021 [72].

**Table 3:** Medicinal plants status according to EFSA legislation [104].

Medicinal Plant	Morphological Element Used in Folk Medicine	Registered as Novel Food
*Morus alba* L. White mulberry	fruits	NO
	young leaves	N/A * but authorized in food supplement use
	stems	N/A * but authorized in food supplement use
	rhizome (root bark)	N/A * but authorized in food supplement use
	root	N/A
	twigs	N/A
*Trigonella* *foenum-graecum* L. Fenugreek	seeds	NO
	leaves	N/A
*Cinnamomum zeylanicum* J.Presl Ceylon cinnamon	bark of the branches	NO
	leaves	NO * only applies to food supplements
	oil of the leaves	N/A
	flowers	N/A
*Phaseolus vulgaris* L. Common bean	seeds	NO
	pods	NO
*Zingiber officinale* Rosc. Ginger	rhizome	NO
*Panax ginseng* C.A.Meyer Ginseng	root	NO
	berries	NO
	leaves	NO
	oil	NO

YES—element has not been used as food in European Union before 15 May 1997 and has a safety assessment status; NO—element has been used as food in European Union before 15 May 1997 and does not need safety assessment; N/A—there was no request or a request has not been processed yet; *—exceptions.

**Table 4:** Some studies considering medicinal plants, as described in this mini review.

Medicinal Plants	Effective Dose, Intake Duration, and Form of Plant Material	Effects of Consumption in In Vivo Models (Level of Change)	Source
*Morus alba* L. White mulberry	20 mg/100 g b.w./d|5 w|leaf extract	R: FBG reduction (5%)	[19]
	0.8 g and 1.2 g|single dose|leaf powder enriched with DNJ (1.5%)	H: PPG_60min_, PPG_90min_ inhibition, insulin secretion inhibition	[99]
	100 mL (1 g of leaves)|tea	R: inhibition of α- glucosidase activity	[100]
	400 mg/kg b.w.|7 w|fruits (polysaccharides)	R: FBG reduction (31.9–47.5%), FSI reduction (3.41–4.19 mIU/L), OGTT reduction (18.12–19.30)	[101]
	6 g/kg b.w.|28 d|leaves with oat bran (1:1)	M: FBG reduction, PPG_60min_ reduction, aspartate transaminase inhibition, alanine transaminase inhibition	[102]
	20 mg and 40 mg and 80 mg/kg|4 w|DNJ extracted from leaves	M: BG reduction, b.w. reduction, SI reduction, HOMA-IR index reduction	[55]
	30 mg/kg b.w.|4 w|morin from leaves	R: downregulation of PERK-eIF2α-ATF4 pathway, BG reduction (69.42%)	[56]
	600 mg/kg b.w./d|6 w| ethanolic leaf extract or leaf powder	R: FBG reduction, TC reduction, TG reduction, LDL reduction; leaf powder more effective than leaf extract	[91]
	2 g/kg b.w./d|4 w|leaf extract	R: FBG reduction, OGTT reduction, HOMA-IR reduction, TC reduction, TG reduction, LDL reduction, insulin resistance improved	[97]
*Trigonella* *foenum-graecum* L. Fenugreek	50 mg/d|30 d|seed powder solution	H: TC reduction (13.6%), TG reduction (23,53%), LDL reduction (23,4%), HDL improved (21.7%)	[1]
	50 mg/kg b.w./d|6 d|4-hydroxyisoleucine	R: BG reduction (from 163.5 mg/dL to 143.6 mg/dL), FSI reduction (from 1.96 ng/mL to 1.52 ng/mL), glucose tolerance improved	[58]
	0.25 g and 0.5 g/kg b.w./d|14 d|ethanolic seeds extract	R: serum glucose reduction (similar to glibenclamide effect), TG reduction, TC reduction, b.w. reduction (5.5% and 9.5%)	[118]
	100 mg/kg b.w./d|4 w|fenugreek extract	R: BG reduction, level of liver enzymes (aspartate aminotransferase and alanine aminotransferase) reduction, TG reduction	[20]
	500 mg/d|30 d or 60 d or 90 d|seed extract enriched with 40% furostanolic saponins	H: FBG reduction (6.69%, 10.31%, 21.98%); PPG_60min_ (13.7%, 20.6%, 30.4%); HbA_1c_ reduction	[119]
	1000 mg/d|12 w|seed extract	H: FBG reduction (24.62%), HbA_1c_ reduction (9.38%); TC reduction (5.66%), TG reduction (17.23%), LDL reduction (4.15%)	[120]
*Cinnamomum zeylanicum* J.Presl Ceylon cinnamon	5 mg and 10 mg and 20 mg/kg b.w./d|45 d |cinnamaldehyde	R: BG reduction (60.8, 139.3 and 219.0 mg/dL)	[62]
	200 mg/kg b.w./w|4 w|ethanolic extract	R: BG reduction (from 257 mg/dL to 122.9 mg/dL), HbA_1c_ reduction (2.51%)	[129]
	1 g/d|90 d| cinnamon supplement	H: PPG_90min_ reduced to 224 mg/dL (with cinnamon) vs. reduced to 270 mg/dL (without cinnamon)	[130]
	3000 mg/d|8 w|cinnamon powder	H: SI reduction (by 12.87 mIU/L), FBG reduction (by 0.45 mg/dL)	[131]
*Zingiber officinale* Rosc. Ginger	25 mg and 50 mg and 100 mg and 200 mg/kg b.w./d|single dose|aqueous extract	R: edemas reduction, NOx reduction similar to indomethacin	[66]
	4 mL/kg b.w./d|6 w|ginger juice	R: flattening BG curve, flattening the insulinemia curve	[148]
	100–500 mg/kg b.w./d|30 d|aqueous ginger extract	R: activity of glycolytic enzymes improved	[149]
	200 mg/kg b.w./d|30 d|ethanolic ginger extract	R: reversed hyperglycaemia, activity of extra-mitochondrial and intra-mitochondrial enzymes improved	[150]
	1800 mg/d|8w|dried ginger	H: BMI reduction (0.54 kg/m^2^), HbA_1c_ (1.11%), FBG (51.15 mg/dL), FSI (7.88 mIU/L), TC (31.10 mg/dL), LDL (17.70 mg/dL)	[154]
	2 g/d|12 w|ginger powder	H: FBG reduction (19,41 mg/dL); HbA_1c_ reduction (0.77%); apolipoprotein B reduction (12.45 mg/dL)	[155]
	3 g/d|12 w|ginger powder	H: serum glucose reduction (19.41 mg/dL), HbA1c (0.77%), SI reduction (1.46 µIU/mL), insulin resistance reduction (16.38); high-sensitive CRP reduction (2.78 mg/dL)	[156]
	2 g/d|8 w|ginger powder	H: SI reduction (13µU/mL), LDL reduction (13.7%), TG reduction (11.7%), HOMA-IR reduction (8.1%)	[157]
*Phaseolus vulgaris* L. Common bean	200 mg and 400 mg/kg b.w./d|28 d|aqueous ginger extract	R: GLUT-4 in skeletal muscles increase	[164]
	100 mg/kg b.w./d|2 w or 4 w|cooked common bean	R: FBG reduction (25% or 35%)	[163]
	50 mg and 100 mg and 200 mg and 250 mg/kg b.w./d|40 d|aqueous bean extract	R: FBG reduction (25% or 50%), TC reduction, TG reduction	[165]
	300 mg/kg b.w./d|single dose|bean	R: dose of glibenclamide reduction to improve glycaemia	[166]
*Panax ginseng* C.A.Meyer Ginseng	1500–3000 mg/d|8 w|vinegar ginseng extract	H: HbA_1c_ reduction (0.29–0.56%), FBG reduction (6.76–21.4 mg/dL) compared to placebo	[187]
	2 capsules/d|8 w|30% of hydrolyzed ginseng extract	H: FBG reduction, PPG_60min_	[188]
	2.7 g/d|4 w|fermented ginseng	H: PPG_120min_ reduction (17.2%), glucose curve flattened (27.4%), no effect on FBG	[189]
	6 g/d|12 w|ginseng	H: no effect on SI level, no effect on insulin sensitivity	[190]
	6 g/d|8 w|ginseng	H: obesity level reduced	[191]

W—weeks, d—days, R—rats, M—mice, H—humans, Rb—rabbits, b.w.—body weight, NOx—nitrogen oxides.

## Data Availability

Not applicable.

## Abbreviations

EFSA	European Food Safety Authority
BMI	body mass index
HbA_1c_	glycated hemoglobin
FBG	fasting blood glucose
TC	total cholesterol
TG	triglycerides
H_2_O_2_	hydrogen peroxide
SI	serum insulin
FSI	fasting serum insulin
ROS	reactive oxygen species
NF-κβ	nuclear factor-κβ
NADH	nicotinamide adenine dinucleotide + hydrogen
HOMA-IR	homeostasis model assessment insulin resistance
GLUT-4	glucose transporter type 4
GLUT-1	glucose transporter type 1
PPG_60min_	postprandial glucose level (60 min)
PPG_30min_	postprandial glucose level (30 min)
PPG_120min_	postprandial glucose level (120 min)
OGTT	oral glucose tolerance test
NOAEL	not observed adverse effect level
DPPH	2,2-diphenyl-1-picrylhydrazyl
ABTS	2,2’-azino-bis(3-ethylbenzothiazoline-6-sulfonic acid)
FRAP	ferric reducing antioxidant power
LDL	low-density lipoprotein
UCP-1	uncoupling protein 1
PPAR-α	peroxisome proliferator-activated receptor alpha
CRP	C-reactive protein
FDA	Food and Drug Administration
